# Sirt3 regulates adipogenesis and adipokine secretion via its enzymatic activity

**DOI:** 10.1002/prp2.670

**Published:** 2020-11-15

**Authors:** Oanh Ma, Truc Le, George Talbott, Tram HoangThao Nguyen, Dorothy Ha, Linh Ho

**Affiliations:** ^1^ California Northstate University College of Pharmacy Elk Grove CA USA

**Keywords:** adipogenesis/adipocytes, adipokines, adiponectin, mitochondrial sirtuins, perilipin 1 (Plin 1), Sirt3

## Abstract

The purpose of this research was to identify if Sirt3 plays a role in marrow adipogenesis and adipokines secretion, especially adiponectin using bone marrow‐derived stroma (ST2) cell model. Sirt3 overexpression leads to a significant increase in adipogenesis compared to controls. The induction of adipogenesis by Sirt3 is associated with increased gene expression of adipocyte markers as well as adiponectin/adipokines. In sharp contrast, the inhibition of Sirt3 exhibited significantly decreased adipogenesis, adipocyte markers, and adiponectin/adipokines compared to the controls. Interestingly, perilipin 1 (Plin 1) expression was decreased in Sirt3 induction but increased in Sirt3 inhibition. One hundred and fifteen mitochondrial acetylated peptides from 67 mitochondrial proteins had lower levels of acetylation in adipocytes induced by Sirt3 overexpression (Sirt3OE) compared to the control. Of the 67 proteins less enriched in acetylation, 22 acetylated proteins were decreased by more than twofold. These proteins are considered potential Sirt3 substrates in adipogenesis. In conclusion, Sirt3 has a novel, important role in modulating adipogenesis and adiponectin/adipokine expression. The connection axis among Sirt3‐adipogenesis‐adipokines was linked to its substrates by mass spectrometry analysis. These findings contribute to the efforts of revealing Sirt3 functions and Sirt3 usage as a potential target for treatment of metabolic homeostasis and diseases including type 2 diabetes.

## Graphical abstract

A novel and critical role for mitochondrial Sirt3 in modulating adipogenesis was uncovered in the study. Sirt3 promotes adipogenesis associated with adipokine secretion. Perilipin 1, interestingly, decreased in Sirt3 induction, but increased in Sirt3 reduction. 67 mitochondrial proteins have been identified as potential Sirt3 substrates in adipogenesis by Mass Spectrometry analysis.

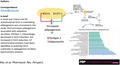

**Abbreviations**
AMPK: AMP‐activated protein kinase
DMSO: dimethylsulfoxide
GDH: glutamate dehydrogenase
HFD: High Fat Diet
IDH: isocitrate dehydrogenase
IL‐6: interleukin‐6, MCP‐1, monocyte chemoattractant protein‐1
IRS‐1: insulin receptor substrate‐1
JNK: c‐Jun N‐terminal kinase
LCAD: long‐chain specific acyl‐CoA dehydrogenase
MDH: malate dehydrogenase
PPAR‐α: peroxisome proliferator activated receptor‐α
ROS: reactive oxygen species
Sirt3KO: Sirt3 knockout
Sirt3OE: Sirt3 overexpression
ST2: Bone marrow‐derived stroma
TCA: tricarboxylic acid cycle
TG: triglyceride

## 1 INTRODUCTION

Metabolic syndrome is also known as syndrome X^1^ and the insulin resistance syndrome.^2^ This condition includes glucose intolerance, insulin resistance, obesity, dyslipidemia, hypertension, and atherosclerosis.^2^, ^3^ Insulin resistance, defined as a relative impairment in the ability of insulin to exert its effects on glucose, protein, and lipid metabolism in target tissues, has many detrimental effects on metabolism and is strongly correlated with the deposition of lipids in non‐adipose tissues.^4^

Adipokines are key regulators of tissue responses to insulin.^5^ Adipocytes have a regulatory role in the development of insulin resistance because they can produce adipokines and because their capacity to store excess lipids can become saturated in obesity, resulting in abnormal redistribution of lipids to other organs and tissues.^6^ Adipose tissues modulate metabolism by releasing non‐esterified fatty acids and glycerol, hormones (including leptin and adiponectin), and proinflammatory cytokines.^7^, ^8^, ^9^ Leptin and adiponectin are anti‐diabetogenic, act as insulin sensitizers, decrease triglyceride (TG) synthesis, stimulate fatty acid oxidation in an AMP‐activated protein kinase (AMPK) and peroxisome proliferator‐activated receptor‐∞ (PPAR‐∞)‐dependent manner.^6^, ^8^, ^10^ Increased release of tumor necrosis factor‐∞ (TNF‐∞), interleukin‐6 (IL‐6), monocyte chemoattractant protein‐1 (MCP‐1), and additional products of macrophages and other cells that populate adipose tissue might also have a role in the development of insulin resistance.^7^, ^11^ In addition, mitochondrial defects trigger activation of several serine kinases, thereby weakening insulin signal transduction.^5^ Thus, elucidating the molecular basis of metabolic syndrome has become one of the most challenging endeavors in modern medicine.^12^

Integrating local and systemic signals with proper mitochondrial function is critical for maintaining metabolic homeostasis. Mitochondrial defects and mitochondrial decline have been observed in metabolic disorders, age‐related metabolic and degenerative diseases, aging, and cancer.^13^, ^14^ Mitochondrial dysfunction in various tissues has been implicated in the pathology of type 2 diabetes mellitus and metabolic syndrome. Reduced mitochondrial oxidative capacity together with increased reactive oxygen species (ROS) generation can increase insulin resistance.^14^

Mammalian sirtuins are nicotinamide adenine nucleotide (NAD)‐dependent protein deacetylases ^15^, ^16^, ^17^ that play crucial roles in major metabolism pathways.^18^ Among the three sirtuins that localize to the mitochondria, Sirt3 is the best‐characterized major mitochondrial deacetylase.^19^ Basal Sirt3 expression varies widely, but the enzyme is highly expressed in metabolically active tissues including liver, kidney, and heart.^20^, ^21^ Sirt3 expression is decreased in mouse models of type 1 or 2 diabetes mellitus.^21^, ^22^ Mice lacking Sirt3 fed a high fat diet (HFD) showed accelerated obesity, glucose intolerance, insulin resistance, hyperlipidemia, and steatohepatitis.^23^ Furthermore, Sirt3‐knockout mice show impaired insulin signaling in skeletal muscle by increased oxidative stress, which led to activation of the c‐Jun N‐terminal kinase (JNK) and decreased insulin receptor substrate‐1 (IRS‐1) signaling following insulin receptor activation.^22^ These findings suggest that Sirt3 plays an important role in diabetes and metabolism, especially fat metabolism, through its deacetylation activity. Surprisingly, both muscle‐specific and liver‐specific Sirt3 knock‐out (Sirt3KO) mice lack overt metabolic phenotypes^24^ suggesting that it is Sirt3 in other tissues that regulate metabolism. Adipose tissue is likely to be critical in mediating the effects of Sirt3 on global metabolism.

Sirt3 has an important role in regulation of metabolic pathways and metabolic diseases such as type 2 diabetes, cancer, and cardiovascular diseases.^25^ However, little is known about the role of Sirt3 in regulating adipogenesis. In a recent report, we ^26^ found that Sirt3 overexpression increased adipogenesis and osteoclastogenesis in bone marrow of aged mice, and produced a corresponding decrease in bone mass. Based on these published data, in the present study, we explored the role of Sirt3 in regulating adipogenesis and adipokines secretion by adipocytes, especially adiponectin using bone marrow‐derived stroma (ST2) cell models. The underlying mechanism of Sirt3 in the regulation of adipogenesis associated with its enzymatic activity was identified. We found that Sirt3, through its deacetylase activity, has a novel, important role in modulating adipogenesis and adiponectin/adipokine expression. The mitochondrial substrates of Sirt3 in the Sirt3‐adipogenesis‐adipokine axis have been identified by mass spectrometry analysis.

## 2 MATERIALS AND METHODS

### 2.1 Transfections

For SIRT3 overexpression (Sirt3OE), ST2 cells were transfected with plasmid DNA of SIRT3‐Myc and empty vector pcDNA 3.1(+) as a control using Lipofectamine according to manufacturer's instructions (Invitrogen, Carlsbad, CA). Lipofectamine‐treated cells were used as a mock control (no DNA). After 24 hours, cells were differentiated into adipocytes by adipocyte differentiation procedure described below.

### 2.2 Sirt3 inhibitor treatment

ST2 cells were treated with Sirt3 inhibitor‐3‐TYP (3‐(1H‐1,2,3‐triazol‐4‐yl) pyridine) (Selleckchem, Houston, Texas) at 50 µM and 100 µM, or equivalent ethanol as control and differentiated into adipocytes by adipocyte differentiation procedure described below. 3‐TYP treatment was applied during the formation of adipocytes (day 1 to day 4), but not during their maturation (day 4 to day 8).

### 2.3 Adipocyte Differentiation

ST2 cells were provided kindly as a gift from Dr Nissenson Laboratory (UCSF) originally obtained from the Riken Cell Bank (Tsukuba, Japan). ST2 cells were differentiated by following the adipocyte differentiation protocol described in Ref. 26 with some modifications. Cells were maintained in growth media: Dulbecco's Modified Eagle Medium (DMEM) supplemented with 10% fetal bovine serum (FBS) and 1% penicillin/streptomycin (P/S). ST2 cell was seeded at a density of 1 × 10^4^/cm^2^ (10^5^ cells/well of six‐well plate) and induced to differentiate by incubation for 2 days with adipogenic differentiation medium (addition of 1 μM rosiglitazone, 1 μM dexamethasone, 5 μg/mL insulin, and 500 μM 3‐isobutyl‐1‐methylxanthine to growth media). After 2 days, this medium was replaced with fresh growth media containing 5 μg/mL insulin for two additional days, and then, cells were maintained in fresh growth media for another 4‐day period. At day 8, cells were fixed in 10% phosphate‐buffered formalin. After fixation, cells were stained with Oil Red O to assess adipogenesis.

Oil Red O dye was also eluted with isopropanol and OD was measured at 500 nm for quantification.

### 2.4 Oil Red O Stain

Oil Red O Staining was performed to measure the amount of stored lipids of the mature adipocytes of the control vs experimental after differentiation. Media were removed from the cells from the six‐well plate and gently washed twice with PBS. For each well, 2 mL of 10% formalin (PERK Scientific, West Chester, PA) was added and incubated for 30 minutes. Formalin was removed and cells were gently washed twice with phosphate‐buffered saline followed by a 5‐minute incubation in 60% isopropanol. The 60% isopropanol was aspirated and 1 mL of Oil Red O solution (Sigma‐Aldrich, St Louis, Missouri, USA) was added evenly over the cells in each well. The six‐well plate was rotated and incubated for 10 minutes. After removing the Oil Red O solution, sterile water was used to wash four times until excess stain was removed. Images were acquired at 250×, and blinded analyses were performed using ImageJ‐analysis software. Oil Red O dye was also eluted with isopropanol and OD was measured at 500 nm for quantification.

### 2.5 Immunoblotting

Differentiated adipocytes were harvested and lysed with RIPA buffer (supplemented with proteinase and phosphatase inhibitors). Protein concentrations were determined by BCA assay. Cell lysates were normalized by concentration and run through an SDS‐Page gel followed by immunoblot analysis with specific antibodies against perilipin 1, pan acetylysine (Cell Signaling Technology), adiponectin Thermo Scientific # PA1‐054), Sirt3 (as described^19^), and β‐actin (A1978, Clone AC‐15, Sigma).

### 2.6 RNA extraction and Real‐time PCR

Mature differentiated adipocytes from ST2 cells were harvested, followed by RNA extraction using RNA STAT60 (Tel‐Test, Inc, Friendswood, Texas) and subsequent purification using PureLink RNA Mini Kit (ThermoFisher Scientific, Waltham, MA USA). cDNA was synthesized using TaqMan Reverse Transcription reagents (Applied Biosystems, Inc, Foster City, California, USA) and random hexamer primers according to the recommendations of the manufacturer. Gene amplification using primers (Table S1) was measured with SYBR Green using the CFX Connect™ Real‐Time PCR Detection System. All reactions were run in triplicate. After data collection, the mRNA copy number of a specific gene in total RNA was calculated with a standard curve generated with serially diluted plasmids containing PCR amplicon sequences and normalized to total RNA with GAPDH as an internal control for assessment adipogenic makers and adipokines.

### 2.7 Triglyceride assay

Cell culture supernatants were collected at certain time points during adipocyte differentiation and triglyceride levels were measured using a colorimetric triglyceride assay kit from Wako (Osaka, Japan). Briefly, supernatants were incubated with reagents from the kit for 5 minutes at 37°C and absorbance at 600 nm was recorded.

### 2.8 LC‐MS/MS analysis

Cell lysates containing 1.5‐2 mg of proteins from differentiated adipocytes were collected for both the Sirt3OE and control condition, and a comprehensive proteomic analysis by LC/MS/MS analysis was performed at the MS and Proteomics Core Facility, Yale University, New Haven, Connecticut, USA. Briefly, differentiated adipocytes from both the Sirt3OE and control condition were rinsed twice with ice‐cold PBS and lysed with RIPA buffer. Cell lysates from Sirt3OE differentiated adipocytes were then digested with trypsin. The resulting peptides were immunopurified using the kit PTMScan® Acetyl‐Lysine Motif [Ac‐K] (Cell Signaling), and the enriched peptides were analyzed by LC/MS/MS to identify protein identifications.

### 2.9 Statistics

**Figure 1:**
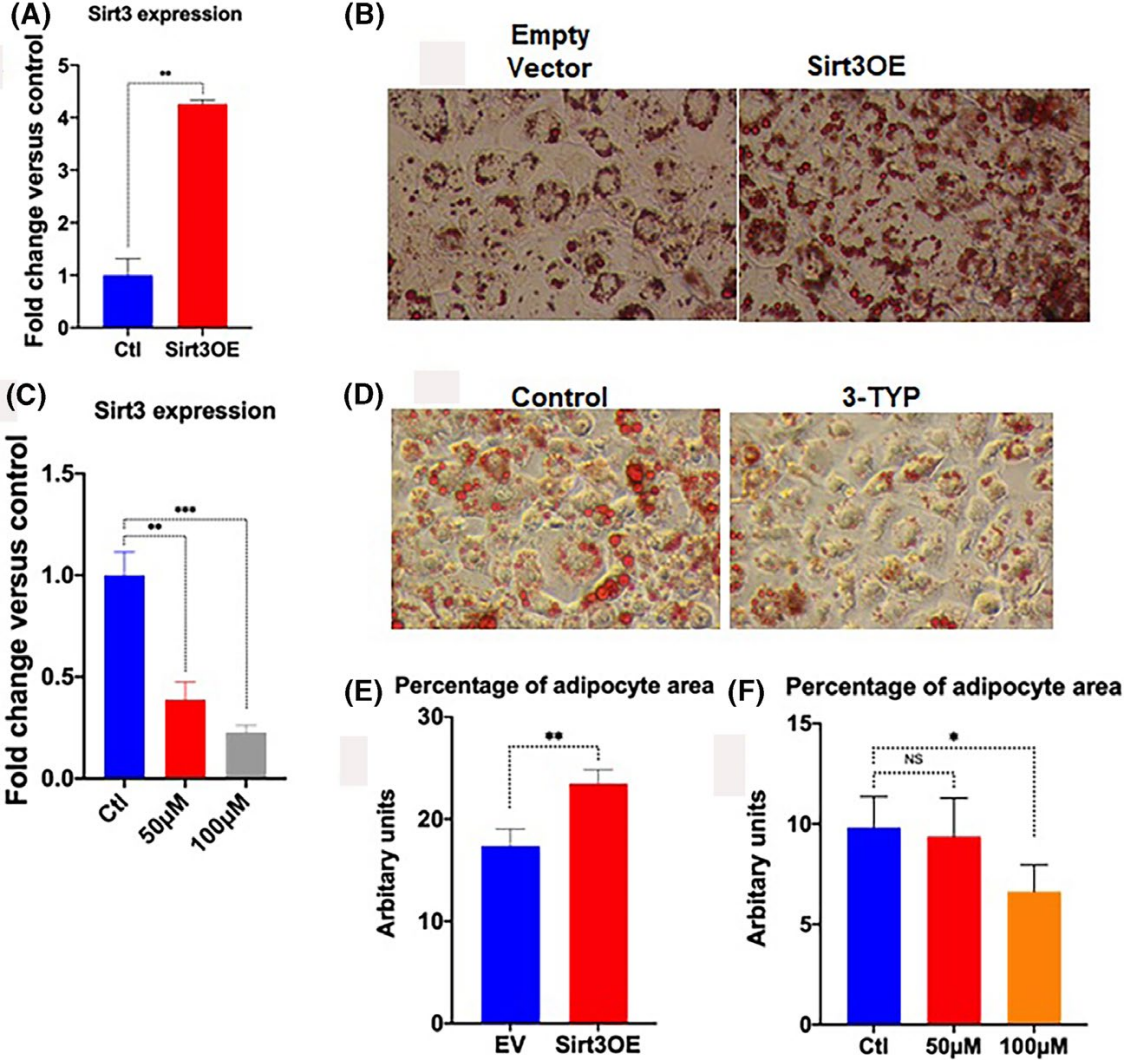
Overexpression of Sirt3 increases adipogenesis, while inhibition of Sirt3 by Sirt3 inhibitor (3‐TYP) decreases adipogenesis. A, Expression level of Sirt3 in Sirt3 overexpression and control in differentiated adipocytes by qPCR; (B) Representative bright‐field images of Oil Red O (ORO) staining with a magnification of 250× of adipogenesis in Sirt3 overexpression vs control in ST2 cells. Adipocytes (lipid droplets) were stained in red color; (C) Expression level of Sirt3 in ST2 cells treated with 3‐TYP at 50 µM and 100 µM compared to control by qPCR; (D) Representative images of adipogenesis in ST2 cells treated with 3‐TYP. Adipocytes (lipid droplets) were stained in red color; Quantification of adipogenesis by percentage of adipocyte area (E) in empty vector (control) and overexpressed Sirt3 in ST2 cells and (F) in cells treated with 3‐TYP at concentrations of 50 µM and 100 µM vs controls. Thirty images of each condition were acquired at 250×, and blinded analyses were performed using ImageJ‐analysis software. Data were collected from at least three independent experiments. Two‐tailed unpaired Student's *t* test with Welch's correction for (A and E), and one‐way analysis of variance analysis (ANOVA) followed by post hoc Dunnette's multiple comparisons for (C and F) were used. *P < .05; **P < .01, ***P < .001; NS: not significant

**Figure 2:**
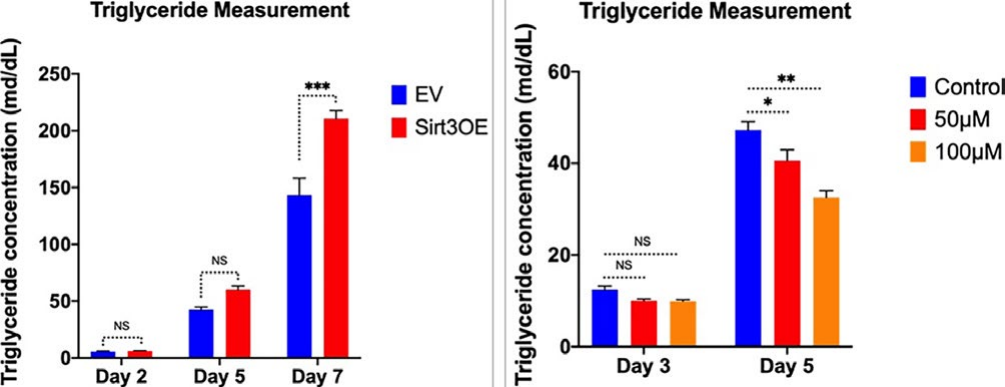
Overexpression of Sirt3 increases triglyceride concentrations, while inhibition of Sirt3 by Sirt3 inhibitor (3‐TYP) decreases triglyceride levels. A, Triglyceride measurements in Sirt3OE vs control (empty vector [EV]) at day 2, day 5, and day 7 after differentiation; (B) Triglyceride concentrations in Sirt3 inhibitor (3‐TYP)‐treated cells at 50 µM and 100 µM vs control, day 3 and day 5 after differentiation. Data were collected from at least three independent experiments. Two‐way ANOVA with Sidak's multiple comparisons was used. *P < .05; **P < .01, ***P < .001

**Figure 3:**
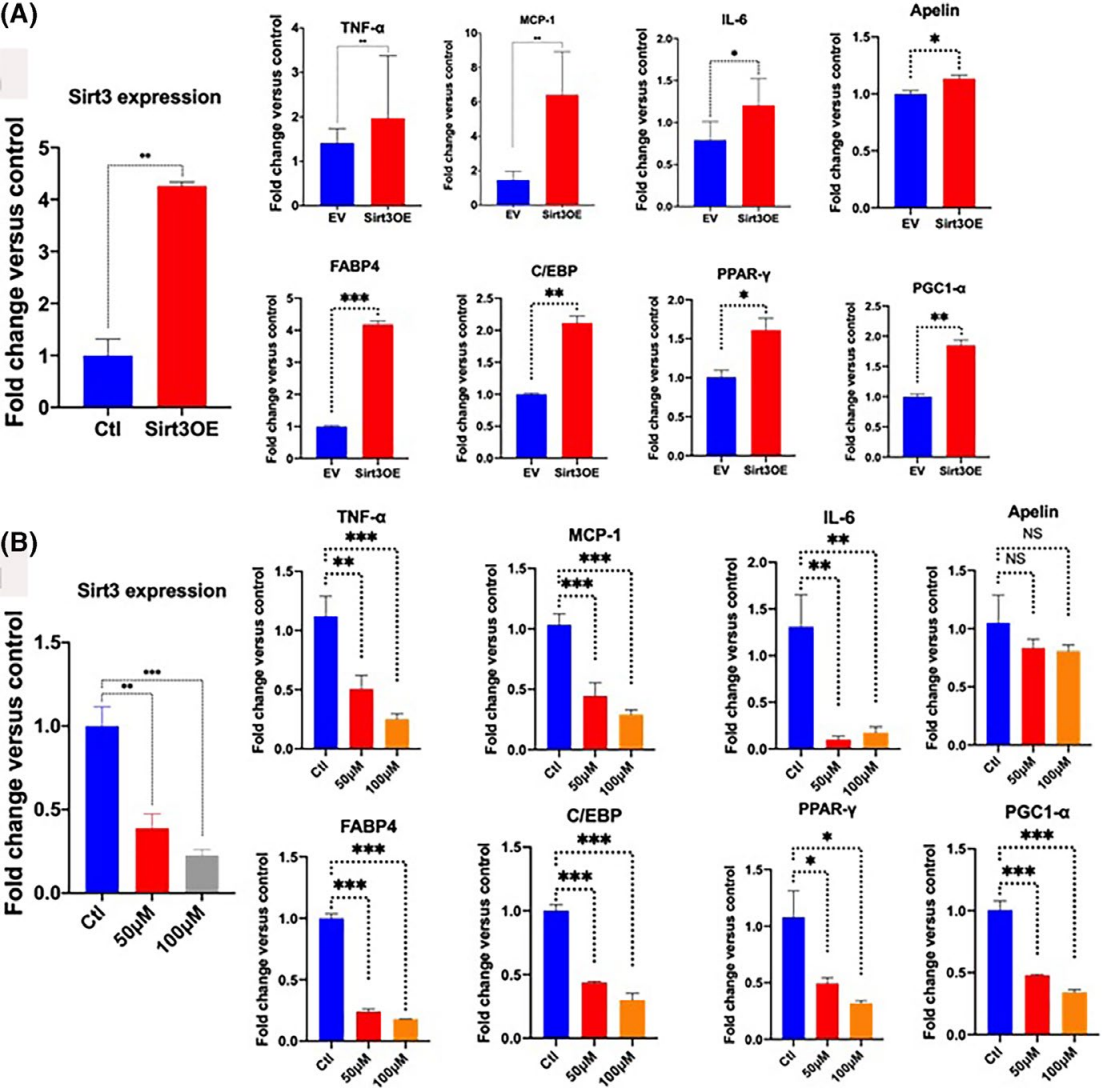
Overexpression of Sirt3 increases adipocyte markers and adiponectin/adipokines, while inhibition of Sirt3 by Sirt3 inhibitor (3‐TYP) decreases adipocyte markers and adiponectin/adipokines. A and B, Expression of Sirt3, adipocyte markers, and adipokine genes in differentiated adipocytes from ST2 cells in Sirt3 overexpression compared to control; C and D, Expression of Sirt3, adipocyte markers, and adipokine genes in differentiated adipocytes from ST2 cells treated with 3‐TYP (Sirt3 inhibitor) at concentrations of 50 µM and 100 µM compared to control. Data were collected from at least three independent experiments. Two‐tailed unpaired Student's t test with Welch's correction was used for Sirt3 overexpressions vs controls, and one‐way analysis of variance analysis (ANOVA) followed by post hoc Dunnette's multiple comparisons for Sirt3 inhibitor‐treated samples compared to controls. *P < .05; **P < .01, ***P < .001

Statistical analyses were performed using a two‐tailed unpaired Student's t test or one‐way analysis of variance analysis (ANOVA) followed by post hoc Dunnette's multiple comparisons or two‐way ANOVA with Sidak's multiple comparisons by Graphpad prism software (version 8.4.2, April 17, 2020, Graphpad Software, La Jolla, CA, United States). For Figure 1, two‐tailed unpaired Student's *t* test with Welch's correction (1A and E) and one‐way analysis of variance analysis (ANOVA) followed by post hoc Dunnette's multiple comparisons (1C and F) were used. For Figure 2, two‐way ANOVA with Sidak's multiple comparisons was used. For Figure 3, two‐tailed unpaired Student's t test with Welch's correction was used for Sirt3 overexpressions vs controls, and one‐way analysis of variance analysis (ANOVA) followed by post hoc Dunnette's multiple comparisons for Sirt3 inhibitor‐treated samples compared to controls. For Figure S1, two‐tailed unpaired Student's t test with Welch's correction (for Sirt3 overexpression vs control) and one‐way analysis of variance analysis (ANOVA) followed by post hoc Dunnette's multiple comparisons (for Sirt3 inhibitor‐treated samples compared to control). Each series of experiments was repeated at least three times. Results are presented as mean ± SEM All the statistical details of the experiments can be found in the figure legends, including number of repetition times. *P*‐values < .05 were considered to indicate statistical significance. Asterisks (*) were used to denote level of statistical significance. *P < .05; **P < .01; ***P < .001.

### 2.10 Nomenclature of targets and ligands

Key protein targets and ligands in this article are hyperlinked to corresponding entries in http://www.guidetopharmacology.org, the common portal for data from the IUPHAR/BPS Guide to PHARMACOLOGY,^27^ and are permanently archived in the Concise Guide to PHARMACOLOGY 2019/20.^28^, ^29^

## 3 RESULTS

### 3.1 Induction of Sirt3 in ST2 cells leads to increased adipogenesis compared to controls: In contrast, inhibition of Sirt3 decreases adipogenesis

Adipocyte differentiation or adipogenesis is a highly controlled process by hormonal and nutritional signals. Understanding the physiological and pathophysiological mechanisms that underlie adipose tissue formation is fundamental for the development of therapeutic approaches to treat obesity and its related diseases. To determine if Sirt3 can cause an effect on adipogenesis, we used a stromal cell line (ST2 cells) that are derived from murine bone marrow. ST2 cells were transfected with an expression vector encoding Sirt3 and differentiated into adipocytes. We found that the overexpression of Sirt3 leads to increased adipogenesis compared to controls by analyzing Oil Red O staining with Image J and measuring isopropanol elution to quantify lipid content (*P* < .001). In contrast, the inhibition of Sirt3 by 3‐TYP leads to decreased adipogenesis compared to control, especially at 100 µM compared to controls (*P* < .05) (Figure 1).

### 3.2 Sirt3 overexpression results in increased triglycerides compared to controls: In contrast, inhibition of Sirt3 decreases levels of triglycerides

In line with induction of adipogenesis, Sirt3 overexpression results in significantly increased triglyceride levels compared to controls at day 5 (60.36 mg/dL compared to 42.45 mg/dL compared to control, *P* < .01) and at day 7 (143.49 mg/dL vs 210.55 mg/dL of the control, *P* < .03) (Figure 2). These data are also consistent with the total average area of differentiated adipocytes, which was significantly higher in Sirt3‐overexpressing ST2 cells than in the controls using Oil Red O staining (426.996 arbitrary units (AU) compared to 576.648 AU of the control, *P* < .006) (Figure S1).

In contrast, we inhibited Sirt3 function in ST2 cells using an inhibitor of Sirt3 called 3‐TYP at two different concentrations of 50 µM and 100 µM. Triglyceride measurements of 3‐TYP‐treated cells vs controls were conducted. Sirt3 inhibition resulted in decreased triglyceride levels in differentiated adipocytes compared to controls (Figure 2) the effect was most notable at 100 µM on day 5 (*P*‐value <.001).

### 3.3 Sirt3 overexpression results in increased adiponectin, adipokines, and adipogenic markers compared to controls: In contrast, inhibition of Sirt3 decreases levels of adiponectin, adipokines, and adipogenic markers

To determine whether induction or inhibition of adipogenesis by Sirt3 is associated with alterations in adiponectin and adipokines, we measured adipokine expression levels in Sirt3OE and in 3‐TYP‐treated differentiated adipocytes using quantitative polymerase chain reaction (qPCR) and western blotting (WB).

**Figure 4:**
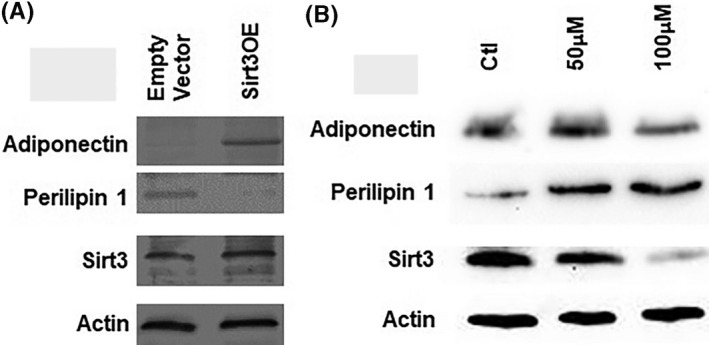
Expression level of adiponectin increases in Sirt3 induction but decreases in Sirt3 inhibition. Interestingly, perilipin 1 decreases in Sirt3 induction but increases in Sirt3 inhibition. A, Expression of adipokine adiponectin and perilipin 1 in differentiated adipocyte from ST2 cells of Sirt3 overexpression and control by WB; actin as loading controls; (B) WB of differentiated adipocytes from ST2 cells treated with 3‐TYP at 50 µM and 100 µM and control against perilipin 1 and adiponectin antibodies, actin as loading controls and Sirt3 as a reference; (C) Expression of Sirt3 gene as reference in differentiated adipogenesis from ST2 cells treated with 3‐TYP (Sirt3 inhibitor) at concentrations of 50 µM and 100 µM compared to control. Data were collected from at least three independent experiments. *P < .05; **P < .01, ***P < .001

The induction of adipogenesis by Sirt3 is associated with increased gene expression of adipocyte markers as well as adiponectin and other adipokines (Figures 3 and 4). In contrast, the inhibition of Sirt3 by Sirt3 inhibitor caused decreased gene expression of adipocyte markers as well as adiponectin and other adipokines (Figures 3 and 4). The expression levels of adipocyte markers and adiponectin/adipokines were statistically significantly reduced compared to the controls in 3‐TYP‐treated cells vs controls (Figures 3 and 4). The reduction of adipokine expression particularly was more pronounced with Sirt3 inhibition. Monocyte chemoattractant protein‐1 (MCP‐1) was the most affected by Sirt3 level in cells. It is significantly increased in Sirt3OE and decreased dramatically in Sirt3 inhibitor‐treated samples compared to controls. In addition, adiponectin expression was induced by Sirt3 overexpression and reduced by Sirt3 inhibition confirmed by both qPCR and WB. Interestingly, the expression level of perilipin 1 was decreased in Sirt3 induction but increased in Sirt3 inhibition compared to controls by WB.

We were able to affirm the role of Sirt3 in modulating adipogenesis associated adiponectin/adipokines.

### 3.4 Identification of potential Sirt3 substrate proteins involved in adipogenesis

Sirt3 is a deacetylase enzyme located in mitochondria. Increase or decrease level of Sirt3 expression will lead to decreased or increased acetylation levels of its protein substrates, respectively, in a complex cellular environment. To understand the molecular mechanism underlying regulation of adipocytes differentiation by Sirt3—a robust deacetylase enzyme, we used mass spectrometry to determine Sirt3 substrates in differentiated adipocytes of Sirt3 overexpression vs controls.

**Figure 5:**
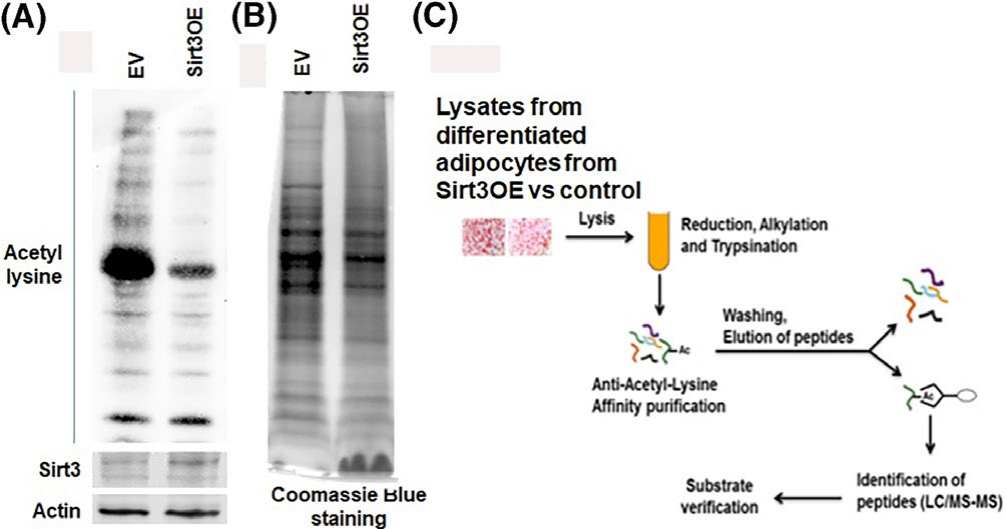
Lysine Acetylation is less enriched in adipocytes induced by Sirt3. (A) WB of differentiated adipocytes from ST2 cells of Sirt3 overexpression and control against pan acetylysine and Sirt3 antibodies, actin as loading controls; (B) Coomassie Blue staining of differentiated adipocyte of Sirt3 overexpression and control. Data came from at least three independent experiments; (C) Strategy for identification of lysine‐acetylated proteins in differentiated adipocytes of Sirt3OE vs control (empty vector [EV]). Cell lysates from differentiated adipocytes from Sirt3 overexpressed ST2 vs control were digested with trypsin. Acetylated peptides were affinity purified using anti‐acetyl‐Lysine before analyzed by LC/MS/MS

A comprehensive proteomic analysis by LC/MS/MS was conducted to identify and compare the acetylated proteins in differentiated adipocytes derived from Sirt3OE vs control. After demonstrating that Sirt3 modulates the acetylation status of proteins in differentiated adipocytes by WB, further identification of the lysine‐acetylated proteins from Sirt3OE compared to control and mapping of the acetylation sites (Figure 5) was conducted. Cell lysates from Sirt3OE‐differentiated adipocytes were digested with trypsin. The resulting peptides were immunopurified using anti‐acetyl‐Lysine antibody (Cell Signaling), and the enriched peptides were analyzed by LC/MS/MS.

### 3.5 Lysine Acetylation is less enriched in adipocytes induced by Sirt3

To detect pan‐lysine acetylation, we used anti‐acetylated lysine antibody to screen for global protein acetylation in differentiated adipocytes of Sirt3OE vs control. We found that the induction of adipogenesis by Sirt3 was associated with Sirt3 enzymatic activity as a robust deacetylase. Acetylation level was reduced in the Sirt3 induction sample compared to control (Figure 5).

### 3.6 Identifying and quantifying the lysine acetylome in adipogenesis induced by Sirt3

To identify acetylated proteins in adipogenesis induced by Sirt3, cell lysates from Sirt3OE‐differentiated adipocytes were digested with trypsin. The resulting peptides were immunopurified using anti‐acetyl‐Lysine antibody (Cell Signaling), and the enriched peptides were analyzed by LC/MS/MS. We searched for potential acetylated proteins that have less acetylation in adipogenesis induced by Sirt3 compared to control.

**Figure 6:**
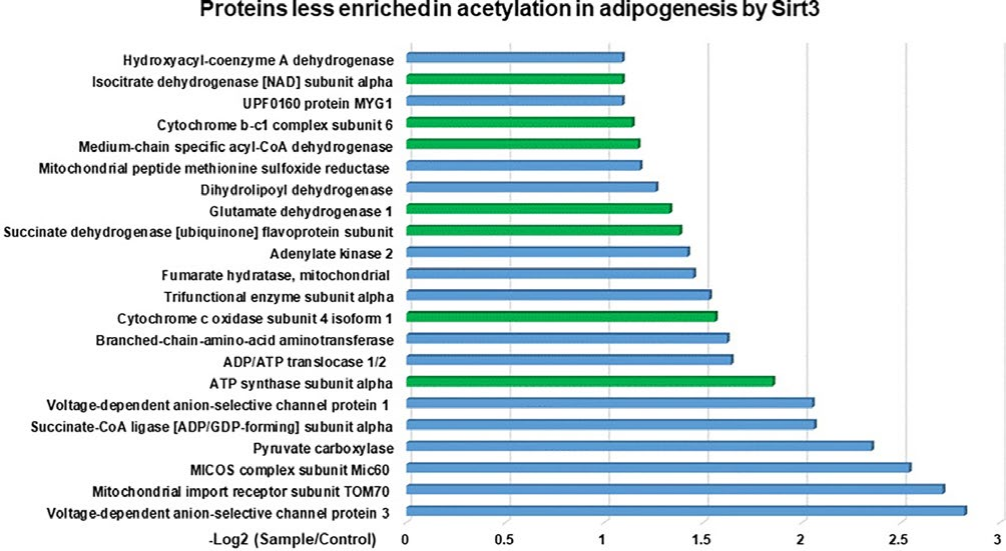
Identifying and quantifying the lysine acetylome in adipogenesis by Sirt3 by LC/MS‐MS. Two mass spectrometry were run on samples from two independent experiments and analyzed. Proteins with less enriched in acetylation (>twofold decrease) in Sirt3OE vs control; Isocitrate dehydrogenase, Cytochrome b‐c1 complex and Cytochrome c oxidase, Medium chain acyl CoA dehydrogenase, Glutamate dehydrogenase, Succinate dehydrogenase, and ATP synthase were demonstrated to be mitochondrial substrates of Sirt3 in previous work from different research groups

We identified 115 mitochondrial acetylated peptides from 67 mitochondrial proteins (Table S4) that have one, two, or three lysine sites with less acetylation in Sirt3OE than that compared to the control. Of the 67 mitochondrial proteins less enriched in acetylation, 22 acetylated proteins (32.83%) were decreased by more than twofold (*P* < .01) (Figure 6 and Table 1). Those proteins with less acetylation in Sirt3OE compared to control are considered as potential Sirt3 substrates in adipogenesis. Among these proteins, ATP synthase, succinate dehydrogenase or complex II, cytochrome c oxidase or complex III, glutamate dehydrogenase, isocitrate dehydrogenase 2, and medium‐chain specific acyl‐CoA dehydrogenase have been found to be Sirt3 substrates regulating metabolism in mitochondria.

**Table 1:** List of acetylated proteins is less enriched in acetylation by more than twofold in adipogenesis induced by Sirt3

Protein Accessions	Protein Descriptions	Sequence	Enrichment Ratio (log2): (Sample)/ (Control)	Acetylation site
Q60931	Voltage‐dependent anion‐selective channel protein 3	YKVCNYGLTFTQK	−2.8	K63
Q9CZW5	Mitochondrial import receptor subunit TOM70	LRPKFALAQAQK	−2.7	K436
Q8CAQ8	MICOS complex subunit Mic60	TSSVTLQTITAQNAAVQAVKAHSNILK	−2.5	K256
Q05920	Pyruvate carboxylase	LDNASAFQGAVISPHYDSLLVKVIAHGK	−2.35	K428
		TLHIKALAVSDLNR		K1041
Q9WUM5	Succinate‐CoA ligase [ADP/GDP‐forming] subunit alpha	GGQKHLGLPVFNTVK	−2.1	K94
Q60932	Voltage‐dependent anion‐selective channel protein 1	GYGFGLIKLDLK	−2.1	K41
		VNNSSLIGLGYTQTLKPGIK		K265
Q03265	ATP synthase subunit alpha	ITKFENAFLSHVISQHQSLLGNIR	−1.85	K506
		FNDGTDEKKK		K241
		HALIIYDDLSKQAVAYR		K316
P48962; P51881	ADP/ATP translocase 1/2	YFPTQALNFAFKDK	−1.64	K94
		VKLLLQVQHASK		K33
		AFFKGAWSNVLR		K272
O35855	Branched‐chain‐amino‐acid aminotransferase	AWIGGVGDCKLGGNYGPTVAVQR	−1.6	K230
P19783	Cytochrome c oxidase subunit 4 isoform 1	DYPLPDVAHVTMLSASQKALK	−1.6	K63
Q8BMS1	Trifunctional enzyme subunit alpha	KYESAYGTQFTPCQLLLDHANNSSKK	−1.53	K760
		RPEKVIGMHYFSPVDK		K493
		ALMGLYNGQVLCKK		K351
	
P97807	Fumarate hydratase	KPVHPNDHVNKSQSSNDTFPTAMHIAAAVEVHK	−1.45	K169
		VLLPGLQKLHDALSAK		K210
		TAHKNGSTLK		K474
Q9WTP6	Adenylate kinase 2	GTQAPKLAENFCVCHLATGDMLR	−1.4	K34
Q8K2B3	Succinate dehydrogenase [ubiquinone] flavoprotein subunit	ACALSIAESCRPGDKVPSIK	−1.4	K480
P26443	Glutamate dehydrogenase 1	TAMKYNLGLDLR	−1.3	K527
O08749	Dihydrolipoyl dehydrogenase	HSAVKALTGGIAHLFK	−1.3	K132
Q9D6Y7	Mitochondrial peptide methionine sulfoxide reductase	VLSKHNFGPITTDIR	−1.2	K183
P45952	Medium‐chain specific acyl‐CoA dehydrogenase	AFTGFIVEADTPGIHIGKK	−1.2	K236
P99028	Cytochrome b‐c1 complex subunit 6	EHCEQLEKCVK	−1.1	K40
Q9JK81	UPF0160 protein MYG1	GGCPWKEHLYHLESELSPK	−1.1	K272
Q9D6R2	Isocitrate dehydrogenase [NAD] subunit alpha	IEAACFATIKDGK	−1.1	K339
Q61425	Hydroxyacyl‐coenzyme A dehydrogenase	TFESLVDFCKTLGK	−1.1	K202

## 4 DISCUSSION

In this study, we demonstrated the novel important role of Sirt3 in modulating adipogenesis and adiponectin/adipokines secretion via its deacetylate activity. The connection axis among Sirt3‐adipogenesis‐adipokines has been linked to its substrates by mass spectrometry analysis.

ST2 cells were chosen because they were derived from long‐term bone marrow culture from BC8 mice, identified as preadipocytes, and serve as a good model for its ability to support insulin‐dependent adipogenesis. By using ST2 cell model, we found that Sirt3 induction caused increased adipogenesis and triglycerides. In contrast, adipogenesis and triglyceride levels were reduced by Sirt3 inhibition in comparison to the controls. This result was consistent with our previous studies showing that Sirt3 is involved in inducing the differentiation of bone marrow stromal cells into different cell lineages^26^ and that at the early stage of adipogenic differentiation, Sirt3 upregulation is essential for the activation of biogenesis and bioenergetic function of mitochondria.^30^

In addition to increased adipogenesis by Sirt3, we demonstrated that this adipogenesis induction was associated with an increase of adiponectin. Conversely, adiponectin was decreased in the Sirt3 inhibition by both qPCR and WB. A decrease in the adiponectin secretion has been associated with insulin resistance in mouse models of altered insulin sensitivity.^31^ It is of great interest to investigate gain and loss of Sirt3 function in adiponectin background mouse models. This result showed that Sirt3 is involved in the regulation of adipogenesis and linked to adiponectin secretion.

We found that the induction of adipogenesis by Sirt3 was associated with increased gene expression of adipocyte markers as well as adipokines. In contrast, the expression levels of adipocyte markers and adipokines were statistically significantly reduced compared to the controls in the inhibition of Sirt3 by Sirt3 inhibitor. Notably, MCP‐1 (monocyte chemoattractant protein‐1) was increased in Sirt3 induction and reduced in Sirt3 inhibition pronouncedly. MCP‐1 regulates the migration and infiltration of monocytes, memory T lymphocytes, and natural killer (NK) cells.^32^ Migration of monocytes from the blood stream across the vascular endothelium is required for routine immunological surveillance of tissues, as well as in response to inflammation.^32^ MCP‐1 also has a major role in promoting mesenchymal differentiation.^32^ This result might suggest that Sirt3 regulates mesenchymal differentiation via increasing adipogenesis linked to induced adipokine‐MCP‐1. Taken together, the induction of adipogenesis associated with increased adiponectin and adipokines secretion in opposite direction with Sirt3 inhibition provides possibly a link to the systemic regulation of metabolism by Sirt3.

Perilipin 1 maintains lipid metabolism homeostasis and regulates the NF‐κB inflammatory pathway in adipocytes.^33^ Perilipin 1 overexpression inhibits the activation of the NF‐κB inflammatory pathway.^33^ It augments the fatty acids and triacylglycerides (TAG) synthesis and inhibiting lipolysis as well as increased inflammatory activation. Sirt3 deficiency has been associated with insulin resistance, obesity, and increase in proinflammatory cytokines and inflammation.^23^, ^34^, ^35^ Interestingly, perilipin 1 expression was increased in Sirt3 inhibition compared to controls in ST2 bone marrow‐derived cells. In sharp contrast, the expression level of perilipin 1 was reduced by Sirt3 induction. This result might suggest that Sirt3 regulates systemic metabolism through adipogenesis induction linked perilipin 1 secretion. It would be critical to further find out the connection of Sirt3‐adipogenesis‐perilipin 1 in mouse model since the target tissues for Sirt3 are yet to be identified. Both muscle‐specific and liver‐specific Sirt3KO mice lack overt metabolic phenotypes.^24^

Sirt3 regulates adipogenesis associated with adiponectin and adipokines secretion through its enzymatic activity, since Sirt3 is a major deacetylase enzyme that is involved in many metabolic pathways in mitochondria. We further identified 67 potential mitochondrial Sirt3 acetylated substrates that were involved in adipogenesis particularly by mass spectrometry analysis. Of these, ATP synthase, succinate dehydrogenase or complex II, cytochrome c oxidase or complex III, glutamate dehydrogenase (GDH), isocitrate dehydrogenase (IDH), aconitate hydratase (aconitase), long‐chain specific acyl‐CoA dehydrogenase (LCAD), and malate dehydrogenase (MDH) have been found to be Sirt3 substrates regulating metabolism in mitochondria in general.

Taken together, adipogenesis requires energy and materials from cellular metabolisms. It does make sense that adipogenesis is also involved in substrates regulated by different metabolic pathways in cells including energy metabolism (such as succinate dehydrogenase or complex II, cytochrome c oxidase or complex III, and ATP synthase), fatty acid oxidation (LCAD), as well as glucose metabolism (such as GDH, IDH, aconitase, and MDH) by deacetylate activity of Sirt3. The mass spectrometry analysis provides us a comprehensive set of possible substrates of Sirt3 in which isocitrate dehydrogenase, cytochrome b‐c1 complex and cytochrome c oxidase, medium chain acyl CoA dehydrogenase, glutamate dehydrogenase, succinate dehydrogenase, and ATP synthase were demonstrated to be mitochondrial substrates of Sirt3 in previous work from different research groups. Since this study identified the first time the set of acetylated proteins in mitochondria that involve in adipogenesis, therefore, they can be considered as proteins of “adipogenesis pathway.”

The beauty of cell models is to get very clear results with a timely manner. However, compared to animal models, cell models might lack of systemic regulation on studied targets of interest.

The study found Sirt3 stimulates adipogenesis using stromal cell line that mimics bone marrow adiposity. It might be interesting to investigate Sirt3 involvement in different adipose tissue depots. The data suggest that adipogenesis is an important process in which Sirt3 yields its effects for regulation of metabolic homeostasis and adipose tissues may be critical sites at which Sirt3 regulates metabolic homeostasis.

## 5 CONCLUSION

For the first time, we identified that Sirt3 induced adipogenesis associated increased adipokines, especially adiponectin and adipokine‐MCP‐1. This induction of adipogenesis resulted from the activation of a subset of Sirt3 substrate candidates such as isocitrate dehydrogenase, cytochrome b‐c1 complex and cytochrome c oxidase, medium chain acyl CoA dehydrogenase, glutamate dehydrogenase, succinate dehydrogenase, and ATP synthase which are involved in energy metabolism, fatty acid oxidation, as well as glucose metabolism. These substrates identified by mass spectrometry can be referred as “lysine acetylome in adipogenesis by Sirt3.” Perilipin 1 was expressed at a higher level during Sirt3 inhibition, but at a lower level during Sirt3 overexpression. A follow‐up study to investigate the Sirt3‐adipogenesis‐perilipin 1 axis would be useful in an attempt to elucidate target cells/tissues of Sirt3.

We hope our study contributes to the efforts of revealing Sirt3 functions in stemness and differentiation as well as metabolic diseases including type 2 diabetes with the ultimate goal of using Sirt3 or its substrate(s) as potential target(s) in the treatment of insulin resistance and other metabolic abnormalities.

## CONFLICT OF INTEREST

The authors have no conflict of interest to declare.

## AUTHOR CONTRIBUTIONS

OM, TL, and GT made substantial contributions to perform experiments and analyze data. THTN and DH performed some experiments and analyzed some data. OM and TL were involved in drafting the manuscript. GT and LH were involved mainly in drafting the manuscript and revising it critically for important intellectual content. LH gave final approval of the version to be published. LH contributed funding support.

## Supporting information

Fig S1

Table S1

Table S2

Table S3

Table S5

## Data Availability

The data that support the findings of this study are available in the supplementary material of this article.
