# Supplementary figures and images for: Sirt3 regulates adipogenesis and adipokine secretion via its enzymatic activity
Source: Pharmacol Res Perspect. 2020 Nov 15;8(6):e00670. doi: 10.1002/prp2.670 (PMC7667394; doi:10.1002/prp2.670)

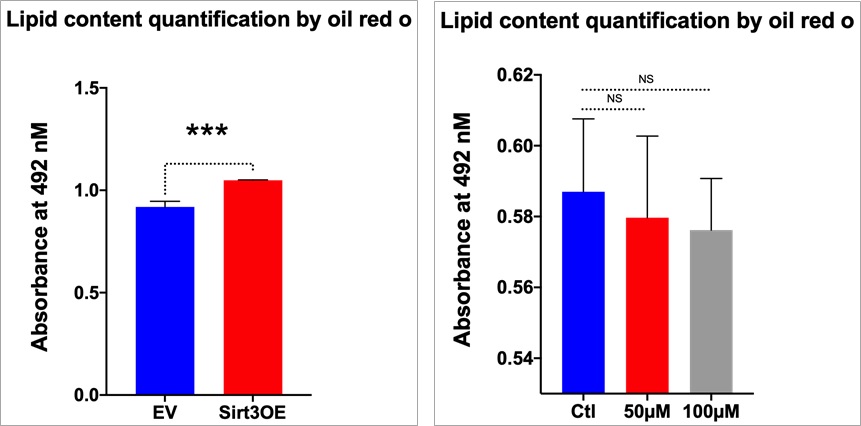

Supplement: Supplementary file 1 — Fig S1 [file PRP2-8-e00670-s001.jpg]
