# Supplementary material for: Sirt3 regulates adipogenesis and adipokine secretion via its enzymatic activity
Source: Pharmacol Res Perspect. 2020 Nov 15;8(6):e00670. doi: 10.1002/prp2.670 (PMC7667394; doi:10.1002/prp2.670)
Supplement: Supplementary file 2 — Table S1 [file PRP2-8-e00670-s002.pdf]

Table S1. List of primers used in the study

Gene	NCBI Ref. Seq. (NM)	Primers	Primer sequences (5'->3')
Leptin	NM_008493	F	CAAGCAGTGCCTATCCAGA
		R	AAGCCCAGGAATGAAGTCCA
Resistin	NM_022984	F	TCACTTTTCACCTCTGTGGATATGAT
		R	TGCCCCAGGTGGTGTAAA
IL6	NM_001314054	F	CCTCTGGTCTTCTGGAGTACC
		R	ACTCCTTCTGTGACTCCAGC
TNFa	NM_001278601	F	ATGAGCACAGAAAGCATGA
		R	AGTAGACAGAAGAGCGTGGT
MCP-1	NM_011333	F	GCTCAGCCAGATGCAGTTAA
		R	TCTTGAGCTTGGTGACAAAAACT
FABP4	NM_024406	F	CAGAAGTGGGATGGAAAGTCG
		R	CGACTGACTATTGTAGTGTTTGA
PPAR gamma	NM_001127330	F	TGATTACAAATATGACCTGAAGC
		R	TTGTAGAGCTGGGTCTTTTCAGAAT
PGC1 alpha	NM_008904	F	TGA TGT GAA TGA CTT GGA TAC AGA CA
		R	GCT CAT TGT TGT ACT GGT TGG ATA TG
C/EBP	NM_007408	F	GTTAGCCATGTGGTAGGAGACA
		R	CCCAGCCGTTAGTGAAGAGT
Apelin	NM_008871	F	GTTGCAGCATGAATCTGAGG
		R	CTGCTTTAGAAAGGCATGGG
