# Supplementary material for: Sirt3 regulates adipogenesis and adipokine secretion via its enzymatic activity
Source: Pharmacol Res Perspect. 2020 Nov 15;8(6):e00670. doi: 10.1002/prp2.670 (PMC7667394; doi:10.1002/prp2.670)
Supplement: Supplementary file 3 — Table S2 [file PRP2-8-e00670-s003.pdf]

Table S2. Triglyceride concentrations (mg/dL) on day 2, day 5, day 7 during adipocyte differentiation of Sirt3 overexpression compared to control (EV) (n = 3/ group).

	EV	Sirt3OE	
	Mean		% induction*
Day 2	5.5551 ± 0.6124	6.167833333 ± 0.2654[A]	11.0301 ± 6.2453
Day 5	42.4522 ± 2.3865	60.3649 ± 2.9528[B]	42.1949 ± 1.3339
Day 7	143.4982 ± 14.6700	210.556 ± 7.2087[C]	46.7307 ± 5.2204

The values are shown as mean ± SEM
* The percentage of induction of triglyceride of Sirt3OE is compared to EV
A: Not Significant (NS): $P = 0.9507$ comparing Sirt3OE and control at day 2
B: Not Significant (NS): $P = 0.0899$ comparing Sirt3OE and control at day 5
C: ****: $P < 0.0001$ comparing Sirt3OE and control at day 7
This analysis used two-way ANOVA with Sidak's multiple comparisons by Graphpad prism software (version 8.4.2, April 17, 2020, Graphpad Software, La Jolla, CA, United States).
