# Supplementary material for: Sirt3 regulates adipogenesis and adipokine secretion via its enzymatic activity
Source: Pharmacol Res Perspect. 2020 Nov 15;8(6):e00670. doi: 10.1002/prp2.670 (PMC7667394; doi:10.1002/prp2.670)
Supplement: Supplementary file 4 — Table S3 [file PRP2-8-e00670-s004.pdf]

Table S3. Triglyceride concentrations (mg/dL) on day 3 and day 5 during adipocyte differentiation of Sirt3 inhibitor treated (50 μM and 100 μM of 3-TYP) compared to control (EV) (n = 4/ group).

	Control	50 μM		100 μM	
	Mean	Mean	% reduction*	Mean	% reduction**
Day 2	12.4825 ± 0.03644	10.0063 ± 0.2021[A]	19.838 ± 2.999	9.8827 ± 0.1857[B]	20.8272 ± 1.4315
Day 5	47.27 ± 0.9229	40.5848 ± 1.1890[C]	14.143 ± 0.5629	32.538 ± 0.7324[D]	31.1656 ± 1.9524

The values are shown as mean ± SEM

* The percentage of reduction of triglyceride of Sirt3 inhibitor treated at 50 μM is compared to control

** The percentage of reduction of triglyceride of Sirt3 inhibitor treated at 100 μM is compared to control

A: Not Significant (NS): $P = 0.5484$ comparing Sirt3 inhibitor treated at 50 μM and control at day 2

B: Not Significant (NS): $P = 0.5484$ comparing Sirt3 inhibitor treated at 100 μM and control at day 2

C: *: $P = 0.0111$ comparing Sirt3 inhibitor treated at 50 μM and control at day 5

C: ****: $P < 0.0001$ comparing Sirt3 inhibitor treated at 100 μM and control at day 5

This analysis used two-way ANOVA with Sidak's multiple comparisons by Graphpad prism software (version 8.4.2, April 17, 2020, Graphpad Software, La Jolla, CA, United States).
